# Clinical efficacy of intra-articular lidocaine with or without adrenaline during shoulder arthroscopy in dogs

**DOI:** 10.3389/fvets.2026.1809857

**Published:** 2026-06-30

**Authors:** Antonello Bufalari, Vicente F. Ratto, Valentina De Monte, Maria Luisa Marenzoni, Giorgia della Rocca, Cecilia Vullo, Tecla Rocchi, Giulia Moretti

**Affiliations:** 1 Department of Veterinary Medicine, University of Perugia, Perugia, Italy; 2 Centro Chirurgico Veterinario Dott. Campanale, CaZampa, Andria, Italy; 3 Department of Chemical, Biological, Pharmaceutical and Environmental Sciences, University of Messina, Messina, Italy

**Keywords:** adrenaline, anesthesia, arthroscopy, dog, lidocaine, pain, shoulder

## Abstract

**Introduction:**

Intra-articular (IA) administration of local anaesthetic drugs has been widely reported in shoulder, elbow, ankle and knee arthroscopy in human medicine. The use of IA adrenaline to control bleeding in arthroscopy is relatively common, but its combined effect with local anaesthetics on perioperative pain has not been studied in dogs. This study aims at evaluating the efficacy of IA shoulder administration of lidocaine or lidocaine plus adrenaline in dogs *(Canis lupus familiaris)* as pre-emptive analgesia during arthroscopic surgery under general anaesthesia.

**Methods:**

Twenty-four dogs were randomly assigned to receive a bolus of lidocaine 2% (group L), a mixture of lidocaine and adrenaline (group LA) or saline solution (NaCl 0.9%) (Control group-group C), injected into a shoulder joint before arthroscopic surgery. Haemodynamic and respiratory parameters were recorded at scheduled times: Baseline (B) at stable anaesthesia and before the painful stimuli; joint injection (J) and 5, 10, 15 minutes post J; trocar insertion (T) and 5, 10, 20, 30, 45, 60 minutes post T. If at least one of the baseline parameters among heart rate, respiratory rate and indirect systolic blood pressure increased by more than 20%, a bolus of 0.2 μg/kg of sufentanil was administered intravenously as rescue analgesia.

**Results:**

Results of this study show that the total dose of sufentanil and the number of boluses required during surgery were significantly lower in group L [odds ratio 6.28 (95% CI: 2-07–18.98)] and LA [odds ratio 8.45 (95% CI: 2.44–29.27)] compared to group C. In group LA, the time of first sufentanil bolus administration was significantly delayed compared to the other two groups. Significant differences in the variability of haemodynamic parameters were observed for HR (*p* = < 0.001), DAP (*p* = 0.041), RR (*p* = 0.013) at T0 (Baseline, before treatment). These findings indicate greater parameter variability in dogs of Group C (control group) compared with the other groups.

**Discussion/Conclusion:**

Compared with placebo, intra articular administration of lidocaine or lidocaine combined with adrenaline just before shoulder arthroscopic surgery in dogs reduces the intraoperative requirement for sufentanil, indicating that this local analgesic approach contributes effectively to a multimodal analgesic protocol.

## 1 Introduction

In human practice, the use of local anesthesia for diagnostic/therapeutic purpose has proved to be rational, useful, cost-effective. However, Intra-articular (IA) administration of local anesthetics is not devoid of risk, and increasing evidence highlights potential adverse side effects, particularly related to chondrotoxicity and joint health, although their clinical relevance remains debated.

Experimental and clinical studies in humans and other species have demonstrated that local anesthetics may induce dose, concentration, and time dependent toxic side effects on chondrocytes, with reported cases of cartilage damage and post arthroscopic chondrolysis following IA administration (36, 38). Although the veterinary literature, particularly in dogs, is less extensive, these findings raise considerations that warrant evaluation when translating IA analgesic techniques across species (37). IA administration has been reported in shoulder, elbow, ankle and knee arthroscopy (1–12, 44).

Even if there are different combinations of agents for IA administration in dogs both for pre-operative and post-operative use (13–17), it is necessary to improve the technique to obtain a pain-free joint without either technical disadvantages or complications due to systemic or local anesthetics toxicity. The application of IA local anesthetics before the beginning of surgery (pre-emptive analgesia) is based on the hypothesis that the neural blockade by local anesthetics prevents the transmission of nociceptive impulses to the central nervous system (CNS) during and immediately after surgery, thereby reducing central sensitization, a condition responsible for the maintenance of post-operative pain (18, 40, 41).

Considerations regarding the pre-emptive use of local anesthetics include the potential for variable pain control, but also systemic toxicity and local adverse effects (19, 20). These risks have been described across multiple species, and although their clinical relevance in dogs remains incompletely defined, they should be considered when evaluating the safety profile of IA administration. Only a few methods of pre-emptive IA administration of LA have been described so far for arthroscopy in dogs, and they differ in volume, concentration, type of local anesthetic, use of adjuvant such as adrenaline and time of local anesthetic instillation (14). The pharmacokinetics of intra-articular lidocaine in dogs have been previously investigated, demonstrating that lidocaine can be used safely in canine arthroscopy, with no signs of systemic toxicity (21). At the same time, recent *in vitro* studies have shown dose- and time-dependent reductions in chondrocyte viability following lidocaine exposure, indicating potential local chondrotoxicity (22).

The use of intra-articular adrenaline to reduce bleeding and improve arthroscopic visualization has been reported in the arthroscopy literature, particularly in human shoulder and hip procedures (23, 43), but its combined effect with lidocaine on perioperative pain has not been studied yet.

In this context, IA administration of local anesthetics may represent a useful adjunct for perioperative analgesia, provided that its efficacy is demonstrated and its safety profile is appropriately considered.

The purpose of this study was to evaluate the efficacy, in terms of pain management, of the IA administration of lidocaine or lidocaine plus adrenaline in dogs undergoing shoulder arthroscopic surgery, within the context of its potential clinical applicability. The hypothesis is that these treatments (both lidocaine alone and lidocaine plus adrenaline) are superior to saline for decreasing intraoperative pain, as attested by a lesser sufentanil administration during surgery.

## 2 Material and methods

The study was conducted by favorable opinion of Bioethical Committee of the University of Perugia (N. 2019-24) and in accordance with the EC Council Directive 86/609 EEC (Council of the European Communities, 1986) adopted by the Italian Laws (Decreto Legislativo n. 116, 1992).

This study was a prospective, randomized, single blind trial. One anesthetist was responsible for IA solution preparation and was aware of group allocation. A second anesthetist, responsible for intraoperative anesthesia management and physiological data recording, remained blinded to treatment allocation. The surgeon and all personnel involved in data collection were blinded to the group assignment. The main surgeon recruited patients requiring shoulder arthroscopy for Osteochondritis Dissecans (OCD) without concomitant ligament or tendon injuries. The procedures were performed by the same surgeon and were limited to arthroscopic removal of cartilage fragment(s) or loose bodies and subchondral debridement.

## 3 Animals and treatments

### 3.1 Animals

Dogs referred to the Veterinary Teaching Hospital of the University of Perugia (Italy) for shoulder arthroscopic surgery due to OCD were enrolled in the study. The sample size was determined based on previous studies with a similar experimental design and outcome measures. In particular, Dutton et al. (13) in a pilot-based sample size calculation for canine arthroscopy, reported that approximately eight joints per group provided adecuate statistical power (80%) to detect clinically relevant differences in intraoperative analgesic requirements under similar experimental conditions. Based on this evidence, the joint was considered the experimental unit, and a comparable group size (*n* = 9 joints per group) was considered appropriate for the present study. Nevertheless, because a small proportion of observations (3/27 joints) were obtained from bilateral procedures in the same dog, complete statistical independence between all observations cannot be assumed, and a limited degree of clustering may have been introduced. Twenty-four dogs were enrolled. Three of these dogs underwent bilateral treatment in two separate surgical sessions, resulting in a total of 27 joints evaluated.

Orthopedic assessment was based on clinical examination and radiographic evaluation of the affected limb. The severity of the articular damage was graded from 1 to 4 (1 = absent, 2 = low, 3 = moderate, 4 = high). Dogs showing capsular swelling associated with extensive synovitis and/or radiographic signs of severe osteoarthritis (OA) (score = 4) were excluded. Additional exclusion criteria included previous local corticosteroid or other drug infiltrations, concomitant systemic anti-inflammatory treatment, or any systemic condition that would preclude safe anesthesia. All enrolled dogs were classified as ASA I based on physical examination and routine pre-operative bloodwork.

### 3.2 Treatments

Dogs were randomly^1^ and single-blindly assigned to one of the three treatment groups according to the pre-surgical intra-articular injection they received: lidocaine (Group L, 9 joints), lidocaine plus adrenaline (Group LA, 9 joints) or saline (Group C, 9 joints).

All dogs underwent anesthesia following a standardized protocol. Premedication consisted of acepromazine maleate (0.01 mg/kg, IM) and methadone (0.2 mg/kg, IM). Thirty to forty min later, anesthesia was induced with propofol (5–6 mg/kg IV) administered through an aseptically placed saphenous venous catheter. After tracheal intubation, dogs were connected to a rebreathing circuit (oxygen flow 30–40 ml/kg) and anesthesia was maintained with isoflurane (1.2–2.0%) in 100% oxygen under spontaneous ventilation. Lactated Ringer's solution was infused at 10 mL/kg/h throughout the procedure. Carprofen (2 mg/kg, SC) and cephalexin (30 mg/kg IV) were administered during patient preparation and at least 120 min before surgery. Anesthetic depth was assessed by evaluating eye position, palpebral reflex and jaw tone, and continuously monitored to ensure that changes in hemodynamic parameters reflected nociception rather that inadequate anesthesia.

Baseline (B) hemodynamic variables: heart rate (HR), indirect blood pressure (SAP, DAP, MAP) measured non-invasively using an oscillometric cuff applied to the forelimb, as well as respiratory rate (RR), end-tidal partial pressure carbon dioxide (EtCO2), hemoglobin oxygen saturation (SpO2) end-tidal isoflurane concentration (EtIso), and rectal temperature (T), were recorded once dogs reached a stable surgical anesthetic plane with EtIso 1.2–1.4, before any nociceptive stimulus. Subsequent hemodynamic measurements were recorded every 5–15 min during surgery, specifically at arthrocentesis and joint distension (J0) (with saline or with lidocaine, combined with adrenaline or not), 5, 10, and 15 min after joint distension (J5, J10, J15), at trocar insertion (T0), and 5, 10, 20, 30, 45, and 60 min thereafter (T5, T10, T20, T30, T45, T60) (Table 1).

**Table 1:** Summary of the data recording phases.

Baseline	J0	J5–J10–J15	T0	T5–T10–T20–T30–T45–T60
Stable plane of surgical anesthesia and before any painful stimuli	Distension of the joint (with saline or with anesthetics)	5–10–15 min after distension of the joint	Trocar insertion	5–10–15–20–30–45–60 min after trocar insertion

A sufentanil bolus (0.2 μg/kg IV over 15–20 s) was administered as rescue analgesia whenever a sympathetic response, defined as an increase of more than 20% above base line HR, RR, or blood pressure, suggested inadequate nociception control. Moreover, adjustments to the anesthetic plan (e.g., isoflurane concentration) were made at the anesthetist's discretion according to individual needs. Rescue analgesia was administered intraoperatively whenever predefined physiological parameters suggested inadequate analgesia, in accordance with ethical and clinical standards to prevent unnecessary pain. Therefore, the administration of a rescue bolus was considered a clinically relevant proxy indicator of intraoperative nociception. Changes in indirect blood pressure (SAP, MAP and DAP), HR and RR were calculated as differences from baseline values.

## 4 Surgical technique

In all patients, the anesthesia was conducted by the same anesthesiology team, and the surgeries were performed by the same experienced surgeon with a standard arthroscopic approach (24). After preparation of the affected limb by trichotomy and cleaning of the surgical area under general anesthesia, each dog was transported in the operating room and positioned in lateral recumbency with the affected limb upward; the skin was aseptically prepared with alcohol and povidone-iodine solution and the surgical field limited with sterile drapes. Arthroscopy was performed following a previously described technique (24). The egress site necessary to aspirate synovial fluid and inject lidocaine, lidocaine plus adrenaline or saline was established first; the needle (20 gauge) was inserted in a cranio- lateral and slight caudal direction, beginning just latero-proximally to the humeral tuberosity, about 0.5 cm distally from the scapular acromion. Synovial fluid was aspirated with a 10 ml syringe attached to the needle (J). Saline (20 ml of NaCl 0.9%), lidocaine (20 ml of lidocaine 2%) or lidocaine plus adrenaline (200 μl of adrenaline (1 mg/ml) to 19.8 ml of lidocaine 2%) solutions were prepared by an assistant in an unlabeled, sterile 20 ml syringe to keep the team unaware of the administered drug. All injections were performed by the same senior surgeon, who injected approximately 0.2–0.4 ml/kg of the prepared solutions; the volume of IA infiltration was judged appropriate on the basis of previous studies (21, 22, 24) and of an adequate joint distention of each shoulder in order to safely insert the trocar into the joint. In any case, the total dose of lidocaine/kg did not exceed 8 mg/kg. Consequently, the dwell time of lidocaine was 15 min. The protocol of the study included also an Intra Lipid Emulsion (ILE) treatment (bolus of 2 mL/kg, IV, over 10 min, followed by a CRI of 4 mL/kg/h, IV) in case of the appearance of systemic side effects due to the local anesthetic. Fifteen min after the IA solution injection (time J15), the second egress site was identified by a needle and, successively, the trocar for the optical instrument was inserted (time T) (24). A 2.7 mm, 30° oblique, rigid arthroscope inside an arthroscopic cannula (Storz) was used to explore the joint. Once arthroscopy started, saline was injected under pressure by an infuser pump bag to maintain a constant and adequate expansion of the joint. The pathology in the joint was identified and scored. A third egress point was performed as well, and the cartilage flap was identified and removed with a pair of arthroscopic forceps as one piece or piecemeal depending on fragment size. Prior to penetration of the subchondral bone (microfracture technique), a meticulous removal of the calcified cartilage layer using an arthroscopic curette or hand burr was mandatory. The microfracture technique (using a pick-like tool) was performed to penetrate the bone surface to allow a releasing of growing factors and to improve healing (25). At the end of the procedure, the portals were closed with cruciate mattress nylon sutures (Ethilon; Ethicon) placed in the skin. Recovery from anesthesia was monitored for the next 24 h. Duration of the whole anesthesia, of the surgical procedure and any perioperative complications were collected.

## 5 Statistical analysis

Data distribution (normality) was assessed using the Shapiro–Wilk test. Baseline comparability between groups (C, L, and LA) for single-measure variables (e.g., body weight, age, anesthesia time, surgery time and time to first rescue analgesia) was evaluated using one-way ANOVA.

Repeated physiological variables (HR, EtCO2, RR, SpO2, SAP, MAP, DAP, EtIso, T), recorded at different intraoperative time points (T0–T60), were analyzed using a general linear model for repeated measures, with treatment group (C, L, and LA) as the between-subject factor and time as the within-subject factor.

When appropriate, *post-hoc* pairwise comparisons between groups and time points were performed using Bonferroni or Tukey adjustments for multiple comparisons.

Rescue analgesia administration (sufentanil bolus) was analyzed as a distinct outcome variable, representing a proxy indicator of intraoperative nociception. The distribution of rescue bolus administration across treatment groups (C, L, and LA) and surgical time intervals (T1–T8) was evaluated using two-way ANOVA as an exploratory analysis.

In addition, logistic regression analysis was performed to assess the association between treatment group (C, L, and LA) and the probability of not requiring rescue analgesia (sufentanil bolus) during surgery. Odds ratios (OR) and 95% confidence intervals (CI) were reported.

Data were analyzed using JSAP^2^ (version 0.16) and R^3^ (version 4.1.0; R Foundation for Statistical Computing, Vienna, Austria). A *p*-value < 0.05 was considered statistically significant.

## 6 Results

Twenty-four dogs (27 shoulder joints) were enrolled in the study. Three dogs (11%) underwent bilateral arthroscopy in separate sessions. The median age was 11.5 months (std 21.2; range 7.0–108.0), and the median body weight was 42 kg (std 12.7; range 12.6–56.0). Six dogs (25%) were male and 18 (75%) were female. The most represented breeds were mixed breed (*n* = 6; 25%), Cane Corso (*n* = 4; 16.6%), Dogue de Bordeaux (*n* = 3 12.5%); Maremma Sheepdog (*n* = 2; 8.3%), and Rottweiler (*n* = 2; 8.3%). One dog (4.2%) belonged to each of the following breeds: Border Collie, Bernese Mountain dog, Boxer, Great Dane, Kurzhaar, Saint Bernard dog, English Setter.

The median anesthesia time was 103 min (range 65.0–170.0), and the median surgery time was 80 min (range 50–135). The median time to the first sufentanil bolus from the beginning of surgery was 8.5 min (range 0.0–45.0). Data on breed, sex, age, IA volume injected, surgery time and anesthesia time stratified by treatment group are summarized in Table 2.

**Table 2:** Patient data: breed, sex, age, surgical and anesthesia time stratified by treatment group.

Data	C (*n* = 9 joints)	L (*n* = 9 joints)	LA (*n* = 9 joints)	*p*
Age (months) (median; range)	10 (7–24)	10 (7–48)	12 (7–108)	0.59
Weight (Kg) (median; range)	40.5 (21–55)	38 (12.6–56)	44 (25–47)	0.83
Breed				0.45
Mixed breed	1 (11%)	3 (33.5%)	2 (22.3%)	
Corso	2 (23%)	3 (33.5%)	1 (11%)	
Dogue de Bordeaux	1 (11%)	1 (11%)	2 (22.3%)	
Maremma Sheepdog	0	0	2 (22.3%)	
Rottweiler	1 (11%)	0	1 (11%)	
English Setter	0	1 (11%)	0	
Saint Bernard dog	1 (11%)	0	0	
Bernese Mountain dog	1 (11%)	0	0	
Boxer	1 (11%)	0	0	
Kurzaar	0	0	1 (11%)	
Border Collie	1 (11%)	0	0	
Great Dane	0	1 (11%)	0	
Sex				0.05
Female	1 (11%)	5 (55%)	1 (11%)	
Male	8 (89%)	4 (45%)	8 (89%)	
Volume injected (ml) (median; range)	16 (8–20)	15 (5–20)	15 (9–18)	0.59
Surgical time (min) (median; range)	70.5 (50–95)	92.2 (60–135)	75.2 (47–105)	**0.048** ^*^
Anesthesia time (min) (median; range)	100 (65–135)	102.3 (80–140)	117.7 (95–170)	0.24

C, control group; L, lidocaine group; LA, lidocaine+adrenaline group; ^*^numbers in bold shows significance.

Data were normally distributed, and parametric tests were therefore applied. The three groups were homogenous with respect to age, weight, sex, and IA volume injected.

All dogs in Group C required at least one sufentanil bolus. The total amount of sufentanil administered was significantly higher in Group C (5.0 μg/kg) compared with Group L (0.8 μg/kg) and Group LA (0.6 μg/kg) (Table 3).

**Table 3:** Variables linked to sufentanil administration stratified for treatment groups.

Variables	C (*n* = 9)	L (*n* = 9)	LA (*n* = 9)	*p*
Time to first sufentanil bolus (min) (mean; range)	15.2; 10–16	2; 0–16 (3 joint only)	11.6; 0–45 (3 joint only)	**0.019** ^*^
Time to second sufentanil bolus (min) (mean; range)	25.7; 16–61	1.7; 0–16 (1 joint only)	None	–
Numbers of sufentanil bolus required (numbers)	24	4	3	**< 0.001** ^*^
Total amount of sufentanil (**u**/kg)	5	0.8	0.6	**< 0.001** ^*^
Mean numbers of sufentanil bolus required for each joint (numbers)	2.7	0.4	0.3	–
Dose of sufentanil required for each joint (μg) (mean ± std)	0.5 ± 0.06	0.09 ± 0.05 (3 joint only)	0.06± 0.03 (3 joint only)	–

C, control group; L, lidocaine group; LA, lidocaine + adrenaline group; ^*^numbers in bold show significances.

**Figure 1:**
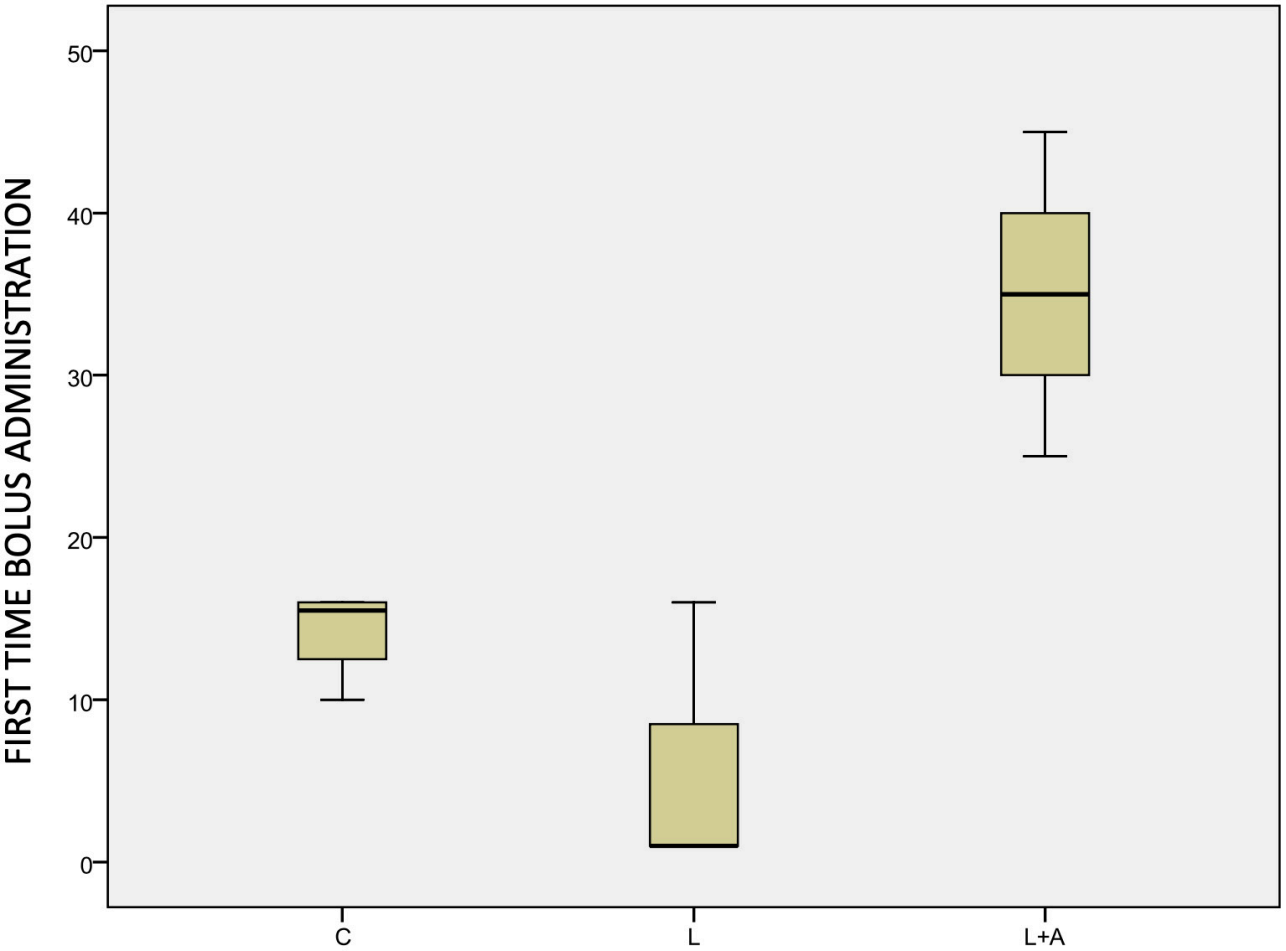
Box plot showing the time of administration of the first bolus of sufentanil in the three groups (C, control group; L, lidocaine group; L + A, lidocaine plus adrenaline group), *p* = 0.019.

**Figure 2:**
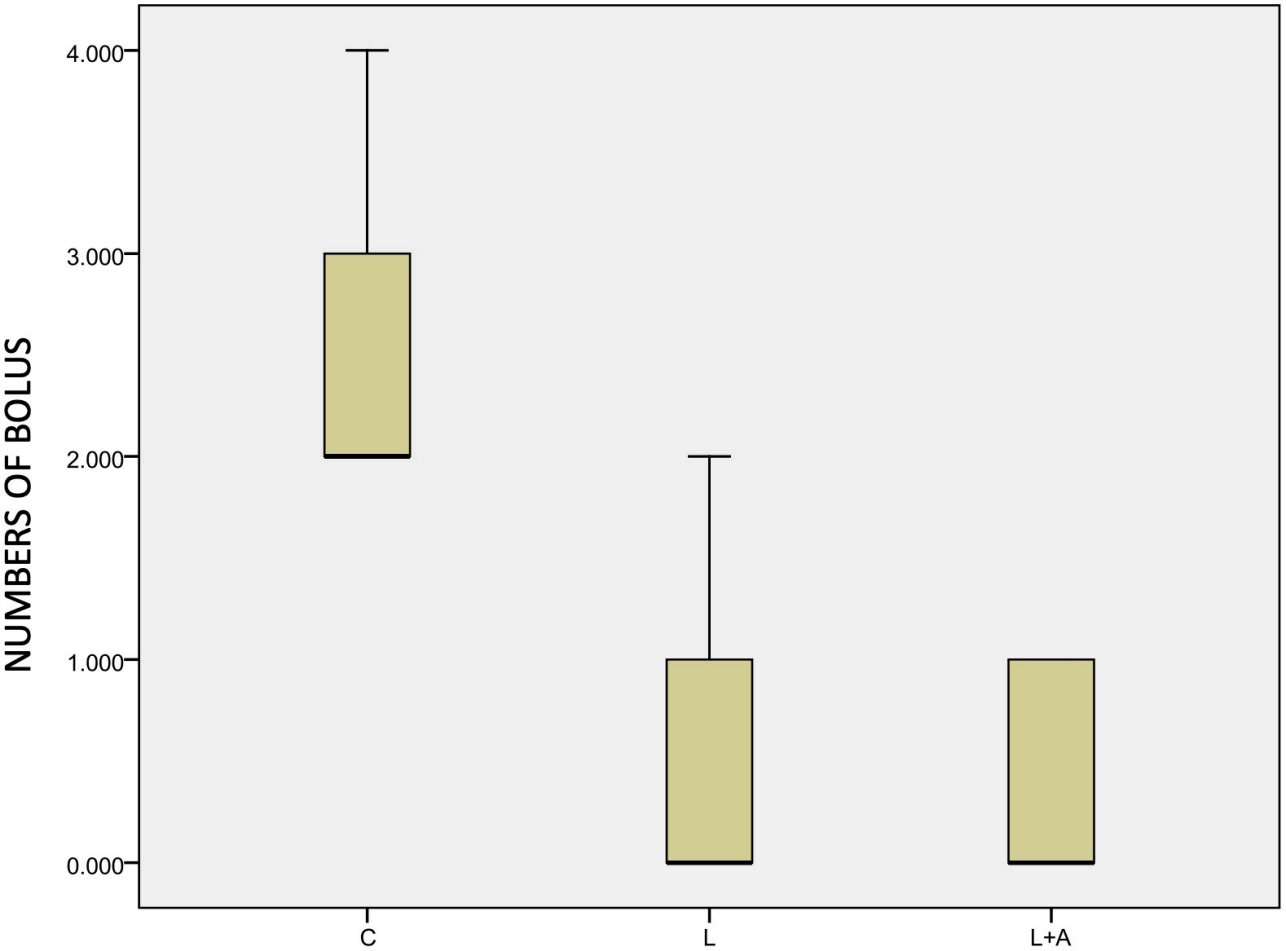
Box plot showing the number of boluses for each treatment group (C, control group; L, lidocaine group; L + A, lidocaine plus adrenaline group), *p* < 0.001.

**Figure 3:**
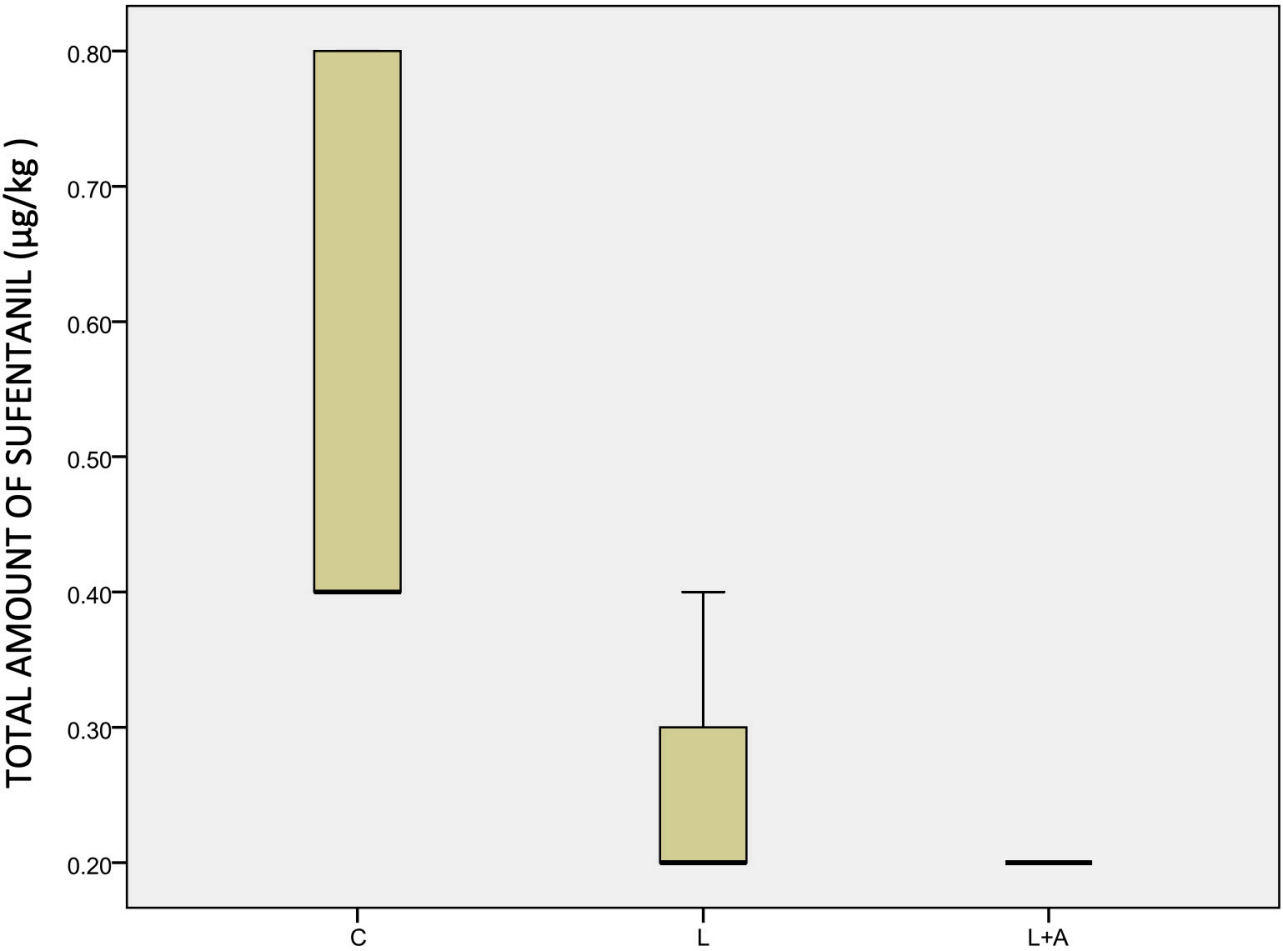
Box plot showing the total amount of sufentanil administered in each treatment group (C, control group; L, lidocaine group; L + A, lidocaine plus adrenaline group), *p* < 0.001.

All variables linked to bolus administration: administration time (*p* = 0.019), number of boluses (*p* < 0.001), and overall amount in μg/kg (*p* < 0.001), were significant between treatment groups. In particular, the interaction between sufentanil boluses and treatment groups, especially with Group C, and the interaction between groups and surgery intervals at time T0, were significant (*p* = 0.032 and *p* < 0.001, respectively). These results suggest that sufentanil boluses were more frequently administered to Group C, particularly during the periods of greatest pain stimuli, such as trocar insertion. No statistical differences were observed in these variables between the groups receiving IA anesthetics (Figures 1–3).

The timing of the first sufentanil bolus varied among groups. In Group L, the first bolus was administered in two dogs 6 min after intra-articular lidocaine injection, just after J5 recording. In Group C, the first sufentanil administration occurred at J10, whereas in Group LA, the first bolus was required at T10. These differences reflect the variable onset of nociceptive responses among groups, with Group LA showing the longest delay before requiring rescue analgesia.

**Figure 4:**
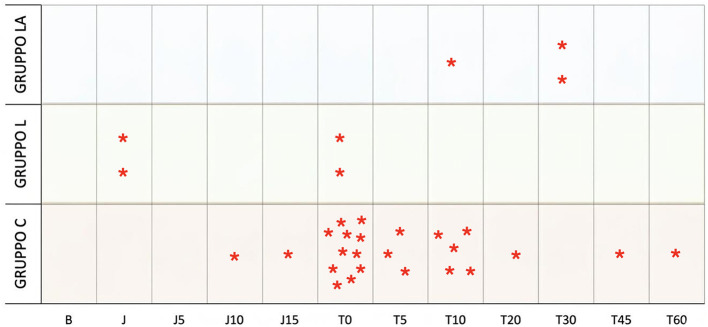
Number of boluses administrated (red asterisk) stratified by treatment groups overtime.

In Group C, eight of the nine dogs (89%) received a bolus at T0; among these, three dogs received two boluses at that same time interval (Figure 4). Nineteen of the 24 boluses administered in Group C (79%) were concentrated within the first three time points following trocar insertion, with 11 boluses (46%) occurring at T0, three boluses (12%) at T5, and five boluses (21%) at T10 (Table 3).

Hemodynamic variability analyses also showed significantly higher variability in HR, DAP, and RR at T0 in Group C compared with Groups L and LA, reflecting greater nociceptive stimulation in dogs receiving saline. Other hemodynamic and anesthetic variables are summarized in Table 4.

**Table 4:** Significant variables stratified for treatment groups.

Parameter	Timepoint	C (*n* = 9)	L (*n* = 9)	LA (*n* = 9)	*post-hoc* comparison	*p*
HR	T0	111 (91–127)	88 (79–98)	91 (90–114)		0.010^*^
					C vs. L	**0.017** ^*^
ETCO_2_	T0	33 (29–46)	36.5 (24–44)	37 (33–42)		**0.024** ^*^
					C vs. LA	**0.022** ^*^
SPO_2_	T45	97(97–99)	97 (97–99)	98 (97–99)		**0.049** ^*^
					L vs. LA	**0.049** ^*^
DAP	J0	41.5 (39–42)	51.5 (37–51)	43.5 (33–55)		**0.049** ^*^
					C vs. L	**0.046** ^*^
	J10	41.5 (37–48)	52.5 (32–72)	39.5 (30–48)		**0.041** ^*^
				–	–	0.780
EtISO	J5	1.2 (1.1–1.3)	1.25 (1.1–1.4)	1.2 (1–1.2)		**0.044** ^*^
					–	0.089
	J10	1.2 (1.2–1.3)	1.2 (1.2–1.4)	1.2 (1–1.2)		**0.041** ^*^
					–	0.094
	J15	1.2 (1.2–1.3)	1.2 (1.2–1.4)	1.2 (1–1.2)		**0.021** ^*^
					C vs. L	**0.045** ^*^
					C vs. LA	**0.045** ^*^
	T0	1.2 (1.2–1.3)	1.2 (1.1–1.5)	1.2 (1–1.2)		**0.044** ^*^
					–	0.060
	T10	1.25 (1.2–1.4)	1.2 (1.1–1.5)	1.2 (0.9–1.2)		**0.013** ^*^
					C vs LA	**0.015** ^*^
	T20	1.2 (1.2–1.4)	1.2 (0.9–1.2)	1.2 (1.1–1.4)		**0.017** ^*^
					C vs. LA	**0.021** ^*^
	T30	1.2 (1.2–1.3)	1.2 (1.1–1.4)	1.2 (1–1.2)		**0.031** ^*^
					C vs. LA	**0.037** ^*^
	T45	1.25 (1.1–1.3)	1.3 (1.1–1.3)	1.15 (1–1.2)		**0.019** ^*^
					C vs. LA	**0.018** ^*^

C, control group; L, lidocaine group; LA, lidocaine +adrenaline group; ^*^numbers in bold show significances.

Hemodynamic variability from baseline was evaluated, revealing significant differences at T0 for HR (*p* < 0.001), DAP (*p* = 0.041), and RR (*p* = 0.013) (Table 5). *Post-hoc* comparisons indicated that variability in these parameters was significantly greater in Group C compared with Groups L and LA.

**Table 5:** Hemodynamic parameters' variability from baseline values between treatment groups.

Parameter	L (*n* = 9)	C (*n* =9 )	LA (*n* = 9)	*p*
HRT0 variability median value (min; max)	4 (−9; 17)	24 (5; 35)	5 (−1; 20)	**< 0.001** ^*^
SAPT0 variability median value (min; max)	9 (2; 29)	2 (−12; 11)	1 (−1; 47)	0.09
MAPT0 variability median value (min; max)	5 (−15; 26)	9 (3; 36)	4 (−2; 19)	0.072
DAPT0 variability median value (min; max)	0 (−8; 23)	10 (0; 31)	1 (−9; 15)	**0.041** ^*^
RRT0 variability median value (min; max)	4 (−1; 12)	10 (0; 29)	2 (−5; 9)	**0.013** ^*^

HRT0, Heart Rate value at Trocar insertion; SAPT0, systolic arterial pressure value at Trocar insertion; MAPT0, mean arterial pressure value at Trocar insertion; DAPT0, diastolic arterial pressure value at Trocar insertion; RRT0, Respiratory Rate value at Trocar insertion; *Post-hoc* comparisons indicated that variability in these parameters was significantly (HRT0 *p* = 0.001, DAPT0 *p* = 0.025, RRT0 *p* =0 .011) greater in Group C compared with Groups L and LA, respectively.

^*^Numbers in bold show significances.

Results from the multivariate logistic regression showed that dogs in Group LA had an 8.45-fold greater odds of not requiring a sufentanil bolus (95% CI: 2.44–29.27), compared with a 6.28-fold greater odds in Group L (95% CI: 2.07–18.98), relative to the control group, demonstrating that intra-articular lidocaine and lidocaine combined with adrenaline both significantly reduced the likelihood of requiring intraoperative rescue analgesia, with Group LA showing the strongest effect. In the control group, the estimated probability of requiring rescue analgesia was approximately 20% of cases, whereas this probability was significantly lower in the treated groups, expressed by the odds ratios for not receiving rescue analgesia.

## 7 Discussion

This prospective, randomized, single-blind clinical trial demonstrated that intra-articular (IA) lidocaine, with or without adrenaline, provides an effective pre-emptive analgesic benefit during shoulder arthroscopy in dogs. Dogs in the control group showed more pronounced nociceptive responses, particularly during the most painful stimuli, such as trocar insertion, whereas dogs receiving IA lidocaine formulations required fewer intraoperative interventions. These findings support the clinical value of IA analgesia as part of a multimodal protocol.

The interpretation of sufentanil administration patterns suggests that IA lidocaine exerts a pre-emptive analgesic effect. The potential influence of adrenaline on this effect remains uncertain and should be interpreted cautiously. However, its use may be pharmacologically justified based on its vasoconstrictive properties, which can reduce systemic absorption of lidocaine, prolong local anesthetic action, and limit peak plasma concentrations, particularly in vascular or inflamed joints (21, 22, 43). However, these mechanisms were not directly evaluated in the present study, and the effects of IA lidocaine combined with adrenaline on joint residence time have not been directly measured in dogs.

Intra-articular (IA) administration of analgesics is widely used in veterinary practice; however, evidence supporting efficacy and safety remains limited and heterogeneous (26, 37, 41). Therefore, IA lidocaine should be considered as part of a multimodal approach rather than a fully validated standalone intervention.

Although continuous saline lavage during arthroscopy may reduce intra-articular drug residence time, an analgesic effect was still observed during key intraoperative stimuli. Post-operative pain was not assessed using valid scoring systems in this study. Routine post-operative follow-up was performed in all cases, including clinical examinations at approximately 14 and 30 days after surgery, as per standard clinical practice. However, the absence of standardized pain scoring limits the ability to draw conclusions regarding post-operative analgesic efficacy. No complications or adverse joint-related outcomes were reported by clinicians or owners during this period, and recovery was consistent with typical cases undergoing shoulder arthroscopy without intra-articular lidocaine. However, longer-term follow-up and objective assessment of joint health were not systematically performed, and therefore subclinical or delayed adverse effects cannot be excluded. These findings are consistent with previous experimental data indicating no evidence of cartilage damage following short-term intra-articular exposure to lidocaine or lidocaine combined with adrenaline under controlled conditions (22, 36, 38, 40, 41).

During shoulder arthroscopy, the main nociceptive stimuli typically include trocar insertion and joint capsule distension. IA injection itself may also induce discomfort (13), and in humans, joint distension is sometimes reported as highly painful (27).

Differences in the timing of the first sufentanil bolus among groups suggest that pain stimuli in the lidocaine-only group may be caused by distension of the inflamed joint capsule, irritation from lidocaine itself, or subtle variations in hemodynamic parameters not observed in the other groups. Although patients with severe joint ectasia and synovitis were excluded, some variability between patients cannot be ruled out.

All dogs received a standardized multimodal pre-operative protocol of methadone in combination with acepromazine and NSAIDs, ensuring baseline analgesia across groups. Physiological variables such as heart rate, respiratory rate, and blood pressure represent indirect indicators of nociception and may be influenced by anesthetic depth and inter-individual variability.

IA lidocaine therefore represented an additional analgesic component beyond systemic treatment (37, 42). No clinically evident adverse events were observed; however, safety was not a primary outcome of this study. Previous studies support the use of local and regional techniques to reduce opioid requirements in canine orthopedic procedures (28, 29), supporting the rationale for incorporating IA lidocaine into multimodal protocols.

The choice of lidocaine in the present study was based on its rapid onset of action, widespread clinical availability, and existing pharmacokinetic data in dogs. However, lidocaine has been associated with dose- and time-dependent chondrotoxicity in several experimental models, and alternative agents such as ropivacaine or mepivacaine may exhibit a more favorable safety profile. These alternatives were not evaluated in the present study due to limited veterinary data in this specific context, but their use warrants further investigation.

The injected solutions were not pH-buffered It is recognized that commercially available lidocaine–adrenaline formulations are acidic, and low pH has been implicated as a contributing factor in chondrotoxicity. Therefore, the potential role of solution acidity, particularly in the lidocaine–adrenaline group, should be considered when interpreting the safety profile of this intervention.

Lidocaine toxicity primarily affects the nervous and cardiovascular systems. In awake dogs, overdose may cause excitement, ataxia, muscle twitches, convulsions, seizures, CNS depression, coma, and eventual respiratory or cardiovascular arrest (30, 31). In this study, no transient ECG changes or bradyarrhythmias were observed and mean arterial pressure remained stable before and after IA lidocaine, indicating that no clinically evident systemic adverse effects were observed at the administered doses. While no clinical or electrocardiographic adverse effects were observed in the present study, the safety of IA local anesthetics should be interpreted with caution. Evidence from human and equine literature indicates that repeated exposure, high concentrations, or prolonged IA residence times may be associated with cartilage injury and, in severe cases, joint degeneration. These findings highlight that the safety of IA local anesthetics is context dependent and influenced by multiple factors, including dose, exposure time, and joint condition.

In this study, the single administration, and short IA dwell time, combined with continuous lavage, likely limited potential chondrotoxic effects. Nevertheless, the possibility of sublinical or long-term joint effects cannot be completely excluded and warrants further investigation in canine patients.

The addition of adrenaline to lidocaine was based on its vasoconstrictive properties, which may influence local anesthetic pharmacokinetics. However, these effects were not directly evaluated in the present study. Experimental studies suggest that local anesthetics may affect chondrocyte viability under certain conditions (32), although clinical relevance remains unclear.

Due to limited literature on IA lidocaine dosing for canine shoulder joints, the volume was based on prior studies in other joints (13, 14, 21, 22, 24). Synovial fluid volume increases in inflamed joints compared to normal dogs (33), and volumes of 5–20 mL of synovial fluid have been reported in dogs with elbow or stifle pathology (34). Accordingly, 0.2–0.4 mL/kg (4–8 mg/kg of 2% lidocaine) was chosen, below the toxic IV dose for anesthetized dogs (35). This volume was considered adequate to safely expand the joint and accommodate individual differences in body weight, inflammation, and capsule distension. Fixed volumes were not used in order to ensure proper joint distention for effective and safe instrument placement, following prior clinical approaches (24).

The prospective, randomized, single-blind design and standardized anesthesia and surgical protocols reduced variability and strengthened internal consistency. While many IA compounds have been studied for intra- or post-operative pain, no studies have directly compared lidocaine vs. lidocaine plus adrenaline in a prospective, randomized, single-blind format.

Despite these factors, several limitations should be acknowledged. The relatively small sample size may have reduced statistical power for some analyses, although pre-study power calculations indicated that nine joints per group would be sufficient to detect clinically relevant differences. In addition, the wide confidence intervals observed in the logistic regression indicate limited precision of the estimated effect size, although the direction of the effect was consistent across groups. Moreover, a small proportion of observations (3/27) derived from bilateral procedures in the same animal, which may introduce a limited degree of clustering. Although the joint was considered the experimental unit, future studies should incorporate mixed-effects models to account for inter individual variability. Furthermore, future investigations could incorporate additional objective outcome measures, such as intraoperative nociceptive reflex monitoring, post-operative pain assessment using validated scoring systems, biochemical stress markers (e.g., cortisol, catecholamines), or continuous hemodynamic monitoring, to enhance the robustness of the findings. Consequently, results should be interpreted with caution and are specifically applicable to young or adult dogs undergoing shoulder arthroscopy under isoflurane general anesthesia within the multimodal analgesic protocol employed in this study.

Nevertheless, this study contributes to the limited evidence on pre-emptive IA lidocaine use in dogs. Direct comparisons between lidocaine and lidocaine–adrenaline require further investigation. It contributes to understanding the efficacy of pre-emptive IA lidocaine, particularly regarding timing of administration. While pre-emptive IA injections have shown potential benefits in human shoulder surgery, few investigations have evaluated pre-emptive IA administration in canine patients, and none have directly compared lidocaine with lidocaine plus adrenaline in a randomized, controlled format.

This study demonstrates that IA lidocaine provides a pre-emptive intraoperative analgesic effect during canine shoulder arthroscopy. The potential additional benefit of adrenaline remains uncertain. These findings support the inclusion of IA lidocaine within a multimodal analgesic protocol.

## Data Availability

The raw data supporting the conclusions of this article will be made available by the authors, without undue reservation.

## Footnotes

1
http://www.randomization.com

2 JASP Team (2021). JASP (Version 0.16) [Computer software].

3 R Development Core Team (2007). Version 2.8.
